# Prospective Study Using Plasma Apolipoprotein A2-Isoforms to Screen for High-Risk Status of Pancreatic Cancer

**DOI:** 10.3390/cancers12092625

**Published:** 2020-09-14

**Authors:** Yu Sato, Takashi Kobayashi, Shin Nishiumi, Akihiko Okada, Tsuyoshi Fujita, Tsuyoshi Sanuki, Masao Kobayashi, Masakyo Asahara, Masayasu Adachi, Arata Sakai, Hideyuki Shiomi, Atsuhiro Masuda, Masaru Yoshida, Keiko Takeuchi, Yuzo Kodama, Hiromu Kutsumi, Kengo Nagashima, Kazufumi Honda

**Affiliations:** 1 Division of Gastroenterology, Department of Internal Medicine, Kobe University Graduate School of Medicine, Kobe, Hyogo 6500017, Japan; satouyuu@med.kobe-u.ac.jp (Y.S.); nishiums@med.kobe-u.ac.jp (S.N.); asakai@med.kobe-u.ac.jp (A.S.); hshiomi@med.kobe-u.ac.jp (H.S.); atmasuda@med.kobe-u.ac.jp (A.M.); myoshida@med.kobe-u.ac.jp (M.Y.); kodama@med.kobe-u.ac.jp (Y.K.); 2 Department of Omics Medicine, Hyogo College of Medicine, Hyogo 6638501, Japan; 3 Department of Gastroenterology, Osaka Saiseikai Nakatsu Hospital, Osaka 5300012, Japan; a.okada@nakatsu.saiseikai.or.jp; 4 Department of Health Care, Yodogawa Christian Hospital, Osaka 5330024, Japan; tsuyofuji@gmail.com; 5 Department of Gastroenterology, Kita-harima Medical Center, Hyogo 6751392, Japan; tssanuki@gmail.com; 6 Department of Health Care, Kyoto Second Red Cross Hospital, Kyoto 6028026, Japan; kobayashim@kyoto2.jrc.or.jp; 7 Asahara Clinic, Hyogo 6730891, Japan; asahara-clinic@fuga.ocn.ne.jp; 8 Hotel Okura Kobe Clinic, Hyogo 6508560, Japan; adachi@okurakobe-clinic.jp; 9 Division of Metabolomics Research, Department of Internal Related, Kobe University Graduate School of Medicine, Hyogo 6500017, Japan; 10 AMED-CREST, AMED, Tokyo 1000004, Japan; 11 Department of Biomarkers for the Early Detection of Cancer, National Cancer Center Research Institute, Tokyo 1040045, Japan; ketakeuc@ncc.go.jp; 12 Center for Clinical Research and Advanced Medicine Establishment, Shiga University of Medical Science, Shiga 5202192, Japan; hkutsumi@belle.shiga-med.ac.jp; 13 Research Center for Medical and Health Data Science, The Institute of Statistical Mathematics, Tokyo 1040045, Japan; nshi@ism.ac.jp; 14 Department of Bioregulation, Graduate School of Medicine, Nippon Medical School, Tokyo 1138602, Japan

**Keywords:** pancreatic cancer, biomarker, cancer screening, apoA2 isoforms

## Abstract

**Simple Summary:**

Apolipoprotein A2 isoforms (apoA2-i) have been identified as minimally invasive biomarkers for detecting pancreatic cancer (PC) and high-risk individuals for PC. We investigated the efficiency of an enrichment strategy for high-risk individuals using a combination of blood testing for apoA2-i with imaging examinations in the general population. We enrolled 5120 subjects in experimental pancreatic cancer screening, with 84 subjects (1.3%) showing abnormal results for apoA2-i. Pancreatic diseases were recognized in about 30% of subjects with an apoA2-ATQ/AT level of ≤35 μg/mL. Among them, 1 pancreatic cancer and 15 high-risk individuals with intraductal papillary mucinous neoplasm were detected. ApoA2-i has the potential to enrich PC and high-risk status by increasing the diagnostic probability before imaging examinations.

**Abstract:**

Apolipoprotein A2-ATQ/AT (apoA2-ATQ/AT) has been identified as a minimally invasive biomarker for detecting pancreatic cancer (PC) and high-risk (HR) individuals for PC. To establish an efficient enrichment strategy for HR, we carried out a plasma apoA2-ATQ/AT level-based prospective screening study among the general population. The subjects for the screening study were recruited at six medical check-up facilities in Japan between October 2015 and January 2017. We evaluated the positive predictive value (PPV) of the plasma apoA2-ATQ/AT level of ≤35 μg/mL for detecting PC and HR. Furthermore, we prospectively confirmed its diagnostic accuracy with another post-diagnosis population in a cross-sectional study. We enrolled 5120 subjects in experimental screening, with 84 subjects (1.3%) showing positive results for apoA2-ATQ/AT. Pancreatic abnormalities were recognized in 26 of the 84 subjects from imaging examinations. Pancreatic abnormalities detected included 1 PC and 15 HR abnormalities, such as cystic lesions including intraductal papillary mucinous neoplasm. The PPV of apoA2-ATQ/AT for detecting PC and HR was 33.3%. Moreover, a combination study with another cross-sectional study revealed that the area under the curve for apoA2-ATQ/AT to distinguish PC from healthy controls was 0.903. ApoA2-ATQ/AT has the potential to enrich PC and HR by increasing the diagnostic probability before imaging examinations.

## 1. Introduction

Pancreatic cancer (PC) is one of the leading causes of cancer-related deaths worldwide [1]. As no advanced techniques have been established to allow early detection and treatment of PC, this pathology has been predicted to become the second leading cause of cancer-related deaths in the United States by 2020 [2]. The five-year survival rate of PC patients with tumors <10 mm in diameter has been reported as 80.4% [3]. Detecting lesions earlier that are smaller in size would reduce the mortality rate of PC. To detect operable earlier stages of PC, the development of efficient methods to screen the general population for PC is essential. On the other hand, the overall lifetime risk of developing PC is approximately 1%, relatively lower than that of lung cancer or colorectal cancer [4]. To establish efficient screening for PC, enrichment with individuals showing a higher prevalence of PC than the general population is needed before invasive and expensive clinical examinations. The most valuable information to select high-risk (HR) individuals is currently a family history of PC. However, we cannot sufficiently detect PC on this basis, because about 90% of PC develops as a sporadic disease. On the other hand, smoking, drinking alcohol, obesity and diabetes are risk factors of PC that are easily identified from medical interviews, physical examinations and simple blood examinations. Unfortunately, the risks associated with these lifestyle factors are not necessarily high when we consider the feasibility of cancer screening. We therefore have to consider enrichment strategies for HR of PC from the general population using the combination of multiple modalities with minimally invasive biomarkers and high-resolution imaging examinations. In cases involving pancreatic diseases such as pancreatic cystic lesions (PCL) [5], including intraductal papillary mucinous neoplasms (IPMN) [6], and chronic pancreatitis (CP) [7], the relative rate of developing PC is higher than in the general population. For example, the incident rate of PC in patients with IPMN is reported as 1% per year [8], and the relative rate of developing PC in patients with CP is 6.9 times higher than in general controls [7]. If those HR diseases or risk factors could be efficiently enriched, close follow-up and preventive interventions may decrease the mortality for PC. Prevalence rates for PCL and CP have been reported as 2.4–3.5% [9,10] and 0.042–0.052% [11,12], respectively. No efficient screening methods have been devised to detect these abnormalities in the general population. Efficient methods thus need to be devised to detect HR and small PC lesions in the general population [13].

In recent years, we have reported apolipoprotein A2 (apoA2) isoforms (apoA2-i) as potential blood biomarkers of the earlier stages of PC [14,15,16]. The clinical performance of apoA2-i has been validated in a prospective cross-sectional multi-institutional study designed by the National Cancer Institute Early Detection Research Network [17] using reference samples collected according to the PRoBE design [18]. In addition, the possible utility of apoA2-i for the risk stratification of PC was also confirmed in a large intracohort case–control study coordinated in Europe [19]. ApoA2 is a major component of high-density lipoproteins, playing an important role in directing lipid metabolism for these lipoproteins [20]. Human apoA2 comprises 77 amino acids, and mainly circulates as a dimer in the bloodstream [20]. The apoA2 protein is classified into three types based on the amino acid sequence of the C-terminus of the monomer: ATQ, AT and A types. This results in five isoforms of the apoA2 homodimer: ATQ/ATQ, ATQ/AT, AT/AT, AT/A and A/A [14]. We have reported that changes in the concentration of apoA2-ATQ/AT due to abnormal processing of the amino acids at the C-terminal end of the apoA2 homodimer could be used to distinguish patients with early-stage PC or HR status from healthy controls [14,15,16]. Therefore, apoA2-ATQ/AT is considered to offer a potentially useful biomarker for screening for PC and HR. However, no previous studies have prospectively evaluated plasma apoA2-ATQ/AT levels as an initial screening tool for PC and HR status in the general population.

Here, we first evaluated the utility of measuring plasma apoA2-ATQ/AT levels as a screening blood test for PC and HR in a prospective study. We also investigated the distribution of plasma apoA2-ATQ/AT levels in the general population. We then assessed correlations between plasma apoA2-ATQ/AT levels and clinical factors. We also calculated the positive predictive value (PPV) of the plasma apoA2-ATQ/AT level for identifying PC and HR status. In addition, in a combination analysis performed as part of an independent prospective cross-sectional study of post-diagnosis patients, we considered the optimal cut-off for plasma apoA2-ATQ/AT levels.

## 2. Results

### 2.1. Overview of the Screening Study

**Figure 1:**
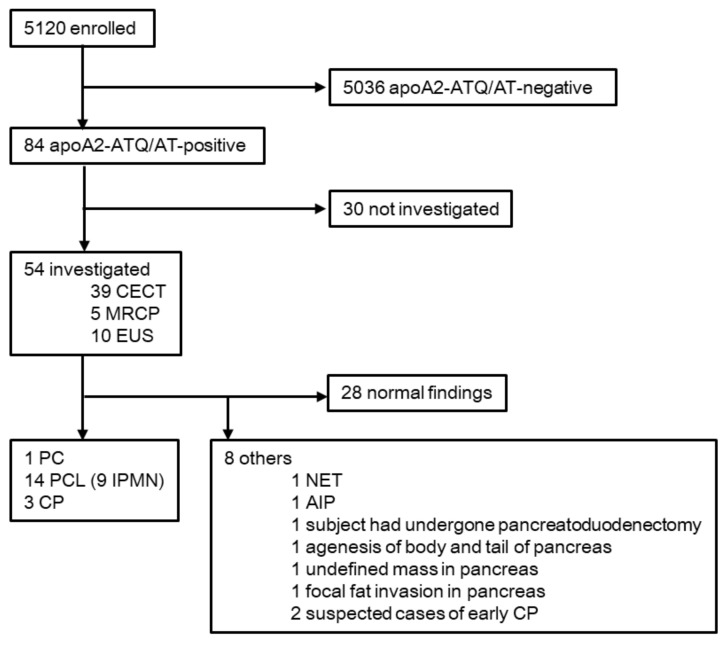
Flow diagram of the screening study. All subjects had plasma apoA2-ATQ/AT levels determined. The apoA2-ATQ/AT cut-off for a positive value was ≤35 μg/mL. CECT, contrast-enhanced computed tomography; MRCP, magnetic resonance cholangiopancreatography; EUS, endoscopic ultrasonography; PC, pancreatic cancer; PCL, pancreatic cystic lesions; IPMN, intraductal papillary mucinous neoplasms; CP, chronic pancreatitis; NET, pancreatic neuroendocrine tumors; AIP, autoimmune pancreatitis.

A total of 5120 subjects were enrolled in the prospective screening study and underwent measurement of plasma apoA2-ATQ/AT levels. The mean (± standard deviation (SD)) plasma apoA2-ATQ/AT level was 62.1 ± 14.4 μg/mL. The distribution of the plasma apoA2-ATQ/AT levels was demonstrated to show an approximately normal distribution (Figure S1). As a result, 84 patients were judged as apoA2-ATQ/AT-positive (plasma apoA2-ATQ/AT level: ≤35 μg/mL). The apoA2-ATQ/AT positivity rate and 95% confidence interval (95% CI) were 1.6% and 1.3–2.0%, respectively. Fifty-four subjects (64.3%) underwent further imaging examinations. As a result, pancreatic abnormalities were detected in 26 of these 54 subjects (48.1%; 95% CI, 34.3–62.2%) (Figure 1).

### 2.2. Background Characteristics of Subjects Enrolled in the Prospective Screening Study

Median age of the 5120 subjects was 52.0 years, with 2662 (52.1%) males (Table 1). Well-known risk factors for PC, including smoking, drinking alcohol, diabetes and high body mass index (BMI), were found to be independent of the plasma apoA2-ATQ/AT level (Table 1). No strong correlations (>0.5, <−0.5) were identified between any of the examined background characteristics and plasma apoA2-ATQ/AT level (Table 1).

### 2.3. Final Diagnosis and Positive Predictive Value for PC and HR Status

As described above, a plasma apoA2-ATQ/AT level ≤35 μg/mL was considered to represent a positive result. Eighty-four of the 5120 subjects exhibited positive plasma apoA2-ATQ/AT results in this study (positivity rate: 1.6%). In a multivariate logistic regression analysis comparing factors that exhibited significance in univariate analyses between patients with positive and negative plasma apoA2-ATQ/AT levels, four factors displayed significance. The apoA2-ATQ/AT-positive group was older and had higher white blood cell counts and lower total cholesterol and low-density lipoprotein levels than the apoA2-ATQ/AT-negative group (Table 2). However, odds ratios for these four factors were not particularly high, at 1.37 (95% CI: 1.01–1.85), 1.32 (95% CI: 1.13–1.56), 0.94 (95% CI: 0.92–0.97) and 1.05 (95% CI: 1.01–1.08), respectively. On the other hand, abnormal pancreatic ultrasonographic findings were detected in 13.3% (95% CI: 6.6–23.2%) of those apoA2-ATQ/AT-positive subjects who underwent transabdominal ultrasonography, compared to only 3.1% (95% CI: 2.7–3.8%) of apoA2-ATQ/AT-negative subjects who underwent transabdominal ultrasonography (*P* = 0.047, multivariate analysis). Thus, the odds ratio for abnormal ultrasonography findings in the apoA2-ATQ/AT-positive group was 4.87 (95% CI: 2.45–9.67). Moreover, the odds ratio for abnormal ultrasonographic findings in the multivariate analysis was 3.04 (95% CI: 1.01–9.14, *p* = 0.047), remaining as a significant independent factor for correlation with reduced apoA2-ATQ/AT.

Fifty-four subjects (64.3% of the apoA2-ATQ/AT-positive group) underwent further screening via imaging examinations. In the apoA2-ATQ/AT-positive group, no significant differences were identified in any of the examined factors between subjects who did and did not undergo imaging, except with regard to aspartate aminotransferase levels (Table S1). Among the 54 subjects who underwent imaging examinations, 39, 5 and 10 underwent contrast-enhanced computed tomography (CECT), magnetic resonance cholangiopancreatography (MRCP) and endoscopic ultrasonography (EUS), respectively. Pancreatic abnormalities were detected in 26 of these 54 subjects (48.1%). The 39 subjects who underwent CECT included 1 subject diagnosed with PC, 8 diagnosed with PCL (including 6 diagnosed with IPMN), CP, agenesis of the body and tail of the pancreas and autoimmune pancreatitis (AIP) in 1 case each, 1 subject who had previously undergone pancreatoduodenectomy and an undefined mass detected in 1 subject. Of the five subjects who underwent MRCP, three IPMN and one case of focal fat invasion were detected. Among the 10 subjects who underwent EUS, 3 PCL, 2 cases of CP, 1 case of pancreatic neuroendocrine tumor (NET) and 2 cases of suspected early CP were detected (Table S2). Of these, 1 PC (stage IV), 14 PCL (including 9 IPMN) and 3 cases of CP were categorized as PC or HR status in this study. Therefore, the PPV of the plasma apoA2-ATQ/AT level for identifying PC or HR status was 33.3% (18/54; 95% CI: 21.1–47.5%) (Figure 1).

A subgroup analysis of those subjects who underwent transabdominal ultrasonography was also performed (*n* = 4165). A total of 65 apoA2-ATQ/AT-positive subjects showed normal ultrasonographic findings. Among these, 38 subjects underwent further imaging examinations, with 8 (21.1%) showing HR status (6 PCL, 2 CP) (Table S3).

### 2.4. Cross-Sectional Study

A total of 105 subjects were enrolled in the cross-sectional study conducted at Kobe University Hospital. These participants included 41 subjects with PC, 24 with PCL, 13 with CP, 13 with AIP, 6 with NET, 1 with solid pseudo-papillary neoplasm, 1 with an undefined pancreatic mass and 6 with normal pancreatic findings (Table 3). The sensitivity of the apoA2-ATQ/AT level (at ≤35 μg/mL, the same cut-off value used in the prospective screening study) for each disease is shown in Figure S2 and Table S4. Detailed data for each stage of PC are shown in Table S4.

### 2.5. Receiver Operating Characteristic (ROC) Curve Analysis of Plasma apoA2-ATQ/AT Level

**Figure 2:**
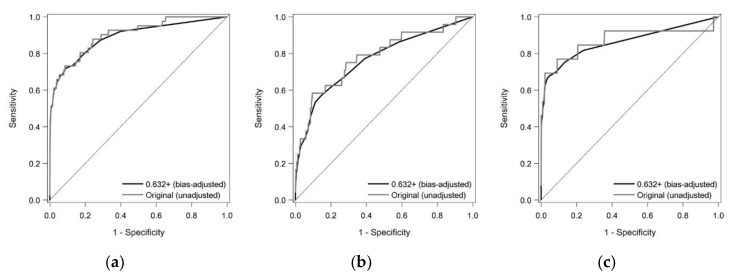
Receiver Operating Characteristic (ROC) curve analysis of the utility of the plasma apoA2-ATQ/AT level for detecting PC and high-risk (HR) status. The gray line shows the original ROC curve, and the black line indicates the bias-adjusted ROC curve created using the 0.632+ bootstrap method: (**a**) the plasma apoA2-ATQ/AT level exhibited area under the ROC curve (AUC) values of 0.889 for detecting PC; (**b**) AUC values of 0.767 for detecting PCL; and (**c**) AUC values of 0.863 for detecting CP.

We performed ROC curve analysis using a combination of subjects from the screening and cross-sectional studies. In the ROC curve analysis, we categorized PC, PCL and CP patients from the cross-sectional study as the abnormal group (*n* = 41, *n* = 24 and *n* = 13, respectively). On the other hand, 4161 subjects from the prospective screening study were used as the control group, based on the definition described in the Methods section. Plasma apoA2-ATQ/AT level exhibited an area under the ROC curve (AUC) value for distinguishing PC patients from healthy controls of 0.903 (95% CI: 0.851–0.955), and the bias-adjusted estimate was 0.889 (Figure 2; Table 4). ApoA2-ATQ/AT level ≤35 μg/mL displayed 51.2% sensitivity (95% CI: 35.1–67.1%) and 98.8% specificity (95% CI: 98.4–99.1%) for detecting PC. The plasma apoA2-ATQ/AT level demonstrated AUC values of 0.864 (95% CI: 0.765–0.963) and 0.930 (95% CI: 0.877–0.983), for detecting resectable and unresectable PC, respectively. The AUC, sensitivity and specificity of the plasma apoA2-ATQ/AT level for detecting PC (resectable or unresectable), PCL or CP at various plasma apoA2-ATQ/AT cut-off values are shown in Table 4.

## 3. Discussion

We have previously identified plasma apoA2-i as a potential blood biomarker of PC [14,15,16]. However, the distribution of plasma apoA2-ATQ/AT levels in the general population has not been clarified. In our prospective screening study, we first demonstrated that the plasma apoA2-ATQ/AT level exhibited an approximately normal distribution in the general population. Therefore, in future studies, researchers can have a high degree of confidence in the validity of the normality assumption when performing statistical analyses. This finding also supports the statistical utility and clinical applicability of ROC curve analysis.

The identification of individuals at high risk of developing PC, such as those with PCL or CP, in the general population is very important for early detection of PC via CECT, MRCP or EUS. In fact, Kenner et al. [13] recommended that using a combination of serological biomarkers and non-invasive imaging is important for defining risk groups for PC and efficiently identifying curable cases of PC among the general population. Kato et al. [21] also proposed a strategy which combines biomarkers with imaging modalities as one of the screening techniques for the early detection of PC. The prevalence of PCL in the general population has been reported to range from 2.4 to 3.5% [9,10]. The risk of PC was demonstrated to be about three times higher in individuals with PCL than in those without it [5]. In particular, among patients with IPMN, two kinds of potential cancer progression need to be considered. One is PC derived from IPMN, and the other is PC that arises concomitantly with IPMN. Various pathways of progression have been reported for IPMN-related invasive PC, some of which involve mutations in KRAS, GNAS or other tumor suppressor genes [22]. Incidence rates of PC derived from and concomitant with IPMN have been reported as 2.2% and 2.0%, respectively, during a 3.7-year observation period [6]. At present, no efficient screening methods exist for detecting PCL among the general population. Our screening study detected 14 PCL (including 9 IPMN) among the 54 apoA2-ATQ/AT-positive subjects who underwent imaging examinations. Furthermore, the PPV of the plasma apoA2-ATQ/AT level for detecting PCL was shown to be 25.9% (95% CI: 14.2–37.6%), about 10 times higher than the prevalence of PCL in the general population. These results indicate that measuring the plasma apoA2-ATQ/AT level as an initial screening method could effectively enhance the detection of PCL. In addition, the prevalence of CP has been reported to range from 0.003 to 0.042% in the general Japanese population [11,12]. The risk of PC has been reported to be 6.9 times higher in patients with CP [7]. In the present study, the PPV of the plasma apoA2-ATQ/AT level for detecting CP was 5.56% (3/54; 95% CI: 0–11.7%), suggesting that measuring the plasma apoA2-ATQ/AT level as an initial screening method could effectively enhance the detection of CP.

Screening the general population for PC is not advisable, because the overall lifetime risk of developing PC is relatively low (approximately 1%). Indeed, PC screening does not meet some of the criteria set by the World Health Organization [4,23]. Information about the accuracy, acceptability, cost and availability of screening tests for PC, and agreement on who should be treated on the basis of the results of such tests are required. As the accuracy of a given test also depends on the prevalence of the disease (pre-test probability), screening for PC is clearly only advisable for specific subpopulations at significantly increased risk of developing the disease. The Cancer of the Pancreas Screening Consortium has stated that PC screening is indicated for some at-risk individuals with a family history of PC or a relevant genetic background [24]. We consider that blood tests of apoA2 should be recommended as an initial screening tool for identifying individuals at high risk of PC among the general population before imaging examinations, such as CECT, MRCP and/or EUS. Detection rates of PCL and PC obtained in the present study based on blood tests of apoA2-ATQ/AT seemed to be similar to those found in previous studies. Blood tests of apoA2-ATQ/AT might thus be useful for efficiently identifying subjects who should undergo imaging examinations, such as CECT, MRCP or EUS. Furthermore, our latest cohort study indicated that PC might be predictable up to 18 months prior to diagnosis by examining levels of apoA2-i [19]. These findings indicate that screening for PC by measuring the plasma apoA2-ATQ/AT level and then performing imaging examinations in appropriate cases could prove effective.

Our screening study demonstrated that a positive plasma apoA2-ATQ/AT level significantly increased the likelihood of detecting abnormal pancreatic ultrasonographic findings. This indicates the utility of measuring plasma apoA2-ATQ/AT levels before transabdominal ultrasonography. However, scanning the whole pancreas using transabdominal ultrasonography within the short period of time available for routine medical check-ups is difficult for large populations [25]. Quality control is another problem with ultrasonography. In this study, PCL or CP was detected during other types of imaging examinations in 21.1% of those apoA2-ATQ/AT-positive individuals in whom no abnormal findings were detected on transabdominal ultrasound (Table S3). We therefore recommend that CECT, MRCP or EUS should be used for further investigations of apoA2-ATQ/AT-positive individuals.

Our latest study reported that biomarkers used to detect PC should provide sensitivity at least 15 times higher than the false-positive rate (e.g., 30% sensitivity with 98% specificity) [19]. In our prospective screening study, the plasma apoA2-ATQ/AT cut-off level was provisionally set at 35 μg/mL. This resulted in bias-adjusted sensitivity and specificity values of 51.3% and 98.8%, respectively, for detecting PC (Table 4). We detected one case of PC among the 54 subjects (1.85%) who underwent imaging examinations after producing positive results during blood tests of plasma apoA2-ATQ/AT levels. This detection rate is not at all disappointing, given the low prevalence of PC in the general population. Moreover, in our ROC curve analysis, the sensitivity of the plasma apoA2-ATQ/AT level for detecting resectable PC was demonstrated to be 41.2% (95% CI: 18.4–67.1%) (40.6% after adjusting for bias) (Table 4). These results seemed to satisfy the abovementioned criterion, even when the disease was at an early, resectable stage, and therefore suggest that the plasma apoA2-ATQ/AT level offers a reasonable initial screening tool.

To reveal the optimal plasma apoA2-ATQ/AT cut-off value for initial screening for PC, we performed further evaluations involving other cut-off values. First, based on Youden’s index, the cut-off value for the plasma apoA2-ATQ/AT level was set at 45.4 μg/mL. This resulted in bias-adjusted sensitivity and specificity values of 72.0% and 90.6%, respectively, for detecting PC. In our cross-sectional study, the plasma apoA2-ATQ/AT level exhibited sensitivity values of 50.0% for stage IA disease, 72.7% for stage IIA disease, 50.0% for stage IIB disease, 81.8% for stage III disease and 76.9% for stage IV disease (Table S4). However, due to the low specificity, the criterion described above was not satisfied. On the other hand, if a false-positive rate of about 5% were to be considered acceptable, the optimal cut-off would be about 40.0 μg/mL. This cut-off value resulted in bias-adjusted sensitivity and specificity values of 61.5% and 96.5%, respectively, for detecting PC. This cut-off value might be more suitable for reducing the risk of overlooking PC without reducing specificity. The performances of the plasma apoA2-ATQ/AT level at each cut-off value are summarized in Table 4.

The underlying reason why the plasma apoA2-ATQ/AT level is useful as a biomarker of PC remains unclear. Five apoA2 isoforms have been identified. We have previously reported that specific abnormal processing patterns of the amino acids at the C-terminal ends of apoA2 homodimers are observed in pancreatic diseases. These processing patterns might be useful for distinguishing PC from CP or AIP, and might also be key to elucidating why the plasma apoA2-ATQ/AT level is useful as a biomarker of PC [26]. Hayasaki et al. [27] recently reported that pancreatic atrophy and insufficient secretion of circulating pancreatic enzymes might influence apoA2-ATQ levels, and suggested that apoA2-ATQ levels could offer a useful biomarker for assessing pancreatic exocrine disorders. As a hypothesis, this abnormal hyper-processing of the C-terminal end of the apoA2 homodimer was anticipated to be caused by the activity of circulating pancreatic enzymes due to minor pancreatitis with pancreatic cancer or other pancreatic disorders, and the abnormal hypo-processing pattern was presumed to occur by reducing the circulating pancreatic enzymes due to pancreatic atrophy and insufficiency.

This study had some limitations that need to be considered when interpreting the results. First, the cut-off value of the plasma apoA2-ATQ/AT level used in the screening study was provisional. Second, actual prevalence rates of PC and other pancreatic diseases in the apoA2-ATQ/AT-negative group could not be evaluated because CECT, MRCP and EUS are too expensive and invasive to perform in all cases. To overcome this problem, we are carrying out another prospective study (UMIN 000028015) which is linked with the Cancer Registry in Japan. Third, the percentage of patients who underwent further examinations was relatively low. Fourth, no gold-standard method has been defined for determining the prevalence of PC. We need to follow-up all subjects for at least a few years to clarify these issues. Fifth, the age criterion used in this study was too wide for a cancer screening study. Sixth, the control group of the ROC analysis was hypothetical.

## 4. Materials and Methods

### 4.1. Study Design

This study was approved by the ethics committee at Kobe University (approval nos. 1716 (approval date 23 April 2015) and 1640 (approval date 20 October 2014)) and was registered with the University Hospital Information Network (UMIN000025857). Written informed consent was obtained from all subjects prior to enrollment in the study. PC was staged according to the Union for International Cancer Control (UICC) classification, 7th edition. All authors had access to the study data and reviewed and approved the final manuscript.

### 4.2. Experimental Prospective Screening Study

We prospectively recruited subjects at 6 medical facilities in Japan from October 2015 to January 2017. All subjects were asymptomatic individuals who were >20 years old and had visited the enrolling medical facility for medical check-ups. Data collected during medical interviews and examinations, such as information regarding age, sex, height, weight, BMI, alcohol consumption, tobacco smoking, medical history, blood counts, blood chemistry and transabdominal ultrasonography findings, were obtained from the medical check-up records of subjects. All blood samples were collected after >8 h fasting, using the standard venous blood sampling method in each facility. Blood count and chemistry were immediately measured in each facility after sampling. Transabdominal ultrasonography was performed by the registered medical sonographer at the same time of blood sampling, and their findings were approved by the physician in each facility. Blood samples for apoA2-ATQ/AT were immediately stored at 4 °C after blood collection, and then the apoA2-ATQ/AT level was measured by a Human APOA2 C-terminal ELISA kit (Toray, Tokyo, Japan) after separating plasma from whole blood. The details of the sample collection for apoA2-ATQ/AT evaluation have been described in the section of Plasma Sample Collection. Measurements of plasma apoA2-ATQ/AT level were performed in a blinded manner at a central laboratory, as described below. The results of the plasma apoA2-ATQ/AT tests were reported to each subject within 3 weeks of blood sampling. In this study, based on the plasma apoA2-ATQ/AT concentrations reported from pancreatic ductal adenocarcinoma patients in a previous study [15], plasma apoA2-ATQ/AT levels ≤35 μg/mL were considered to indicate a positive result. Only those subjects who exhibited positive results were referred for further screening involving imaging examinations by oral announcement or mail. From an ethical point of view, further examinations for the subjects with a positive result was not mandatory. The decision whether to undergo further examinations was based on an honest explanation and recommendation. These imaging examinations were conducted at high-volume centers associated with the medical check-up facilities, and were performed using CECT, MRCP or EUS, depending upon the decisions of the attending gastroenterologists at each facility. If pancreatic abnormalities were detected during imaging examinations, additional detailed examinations and treatment were performed, as would be the case for ordinary patients with appropriate clinical indications. Based on these results and other data from subjects, the final diagnosis for each case was determined by a gastroenterologist at the relevant institution. No further interventions were conducted in the cases in which subjects displayed negative apoA2-ATQ/AT blood test results.

The primary outcome measured in this study was the PPV of the plasma apoA2-ATQ/AT level for detecting PC or HR. We also investigated the distribution of plasma apoA2-ATQ/AT levels in the general population, and correlations between plasma apoA2-ATQ/AT level and well-known risk factors for PC. In addition, the background data of apoA2-ATQ/AT-positive and -negative groups were compared. In the apoA2-ATQ/AT-positive group, differences between subjects who did and did not undergo imaging were also evaluated to reveal potential selection biases.

### 4.3. Cut-Off Value of the Plasma apoA2-ATQ/AT Level

As this was the first prospective screening study to examine the utility of the plasma apoA2-ATQ/AT level as a predictor of PC or HR status in the general population, we set a strict apoA2-ATQ/AT cut-off value to suppress the false-positive rate. In our previous study, mean plasma apoA2-ATQ/AT level in PC patients was 36.6 μg/mL. On the other hand, the mean and standard deviation (SD) in healthy controls were 66.7 and 12.7 µg/mL, respectively [15]. If the plasma apoA2-ATQ/AT level distribution follows a normal distribution in the general population, an apoA2-ATQ/AT level of <37.49 μg/mL would be located around 2.3 SD below the mean (in about the bottom 1.07% of the distribution). In this study, plasma apoA2-ATQ/AT concentrations ≤35 μg/mL were considered to indicate a positive result. The apoA2-ATQ/AT positivity rate in the general population was expected to be about 1%.

### 4.4. Cross-Sectional Study

To determine the most effective cut-off value for this purpose, we designed a prospective cross-sectional observational study. We prospectively recruited post-diagnosis patients who visited the Department of Gastroenterology of Kobe University Hospital between April 2015 and January 2017. Plasma samples and clinical data were obtained from all subjects. Plasma apoA2-ATQ/AT levels were measured in the same manner as in the prospective screening study. These results were not reported to the subjects or their attending doctors. The clinical parameters assessed in this study were the same as those evaluated in the prospective screening study. The sensitivity of the plasma apoA2-ATQ/AT level for detecting various pancreatic diseases was also evaluated, and ROC curve analysis was performed, as described below.

### 4.5. ROC Curve Analysis

The optimal plasma apoA2-ATQ/AT level cut-off values for PC, PCL and CP were investigated via ROC curve analysis. Those cross-sectional study subjects with PC, PCL or CP were included in the ROC curve analysis. The control group from PC and its HR individuals are hypothetically definite. The subjects of the prospective screening study who exhibited normal pancreatic ultrasonographic findings and had no history of pancreatic disease were used as the control group. Subjects who did not undergo transabdominal ultrasonography as part of the medical check-ups were excluded from the ROC curve analysis.

### 4.6. Plasma Sample Collection

All blood samples were obtained after the subjects had fasted for >8 h using the standard venous blood sampling method. Samples were transferred into blood-collecting tubes, which contained ethylenediaminetetraacetic acid-2Na as an anticoagulant. After gentle mixing, collected blood samples were immediately placed in a portable blood tube cooler (CubeCooler; Forte Grow Medical Co., Tochigi, Japan) until centrifugation. The plasma was obtained by subjecting the blood samples to centrifugation at 2270× *g* at 4 °C for 15 min within 8 h of blood sampling, and all plasma samples were stored in −80 °C until analysis.

### 4.7. Measurement of Plasma apoA2-ATQ/AT Level Using an Enzyme-Linked Immunosorbent Assay (ELISA) Kit

Plasma apoA2-ATQ/AT concentrations were measured in a blinded manner at a central laboratory (SRL, Tokyo, Japan). The human apoA2 C-terminal ELISA kit (Toray Industries, Tokyo, Japan) was used to measure levels of each apoA2-i, according to the instructions from the manufacturer. This ELISA kit can be used to measure levels of apoA2-AT and apoA2-ATQ. A previously reported formula based on ELISA results for apoA2-AT and apoA2-ATQ was used to calculate the plasma apoA2-ATQ/AT level in this study [15]. Measurements were taken from distinct samples and the reliability of this ELISA kit has been confirmed in our previous report [15].

### 4.8. Statistical Analysis

Summary statistics are reported as frequencies and proportions for categorical data, and as mean ± SD or median and range values for continuous variables. Patient characteristics were compared using Fisher’s exact test for categorical outcomes and Welch′s t-test or the Wilcoxon rank-sum test for continuous variables, as appropriate. Kernel density estimation was used to estimate the plasma apoA2-ATQ/AT level distribution. Spearman’s correlation coefficients were calculated for the relationships between background characteristics and plasma apoA2-ATQ/AT level. Uni- and multivariate logistic regression analyses were performed to identify variables that could be used to classify subjects according to plasma apoA2-ATQ/AT levels (≤35 μg/mL for a positive result, >35 μg/mL for a negative result). ROC curve analyses were performed to assess the diagnostic accuracy of the plasma apoA2-ATQ/AT level for diagnosing various pancreatic diseases, and the 0.632+ bootstrap method (on 1000 bootstrap samples) was applied to reduce overfitting and optimism. AUC, sensitivity and specificity values for the plasma apoA2-ATQ/AT level were calculated at the prespecified cut-off value of 35 μg/mL and a second cut-off value of 40 μg/mL, achieving the target specificity of ≥95%. Sensitivity and specificity values for the optimal threshold that maximized the sum of sensitivity and specificity were also calculated based on Youden’s index. All tests were two-tailed, and statistical significance was defined as *p* < 0.05. All analyses were performed using SAS version 9.4 software (SAS Institute, Cary, NC, USA) or “R” version 3.5.2 software (R Core Team, Vienna, Austria).

## 5. Conclusions

In conclusion, this prospective study revealed that measuring the plasma apoA2-ATQ/AT level as an initial screening method before performing imaging examinations may enhance the detection of PC and HR status in the general population. To confirm these results, a large-scale interventional study, involving the screening of individuals >50 years old under a combination of measuring blood apoA2-i levels and CECT, was started in Japan in 2018.

## Figures and Tables

**Table 1:** Background characteristics of all subjects in the screening study.

Characteristics	All Subjects in the Screening Study (*n* = 5120)		
	*n*	Values	Spearman’s Correlation Coefficients
Age (years) *	5108	52.0 (22–89)	−0.0775
Sex—males (%) **	5108	2662 (52.1%)	−0.0418
BMI (kg/m^2^) *	4486	22.5 (13.2–44.2)	0.0115
Smokers (%) **	4021	1548 (38.5%)	0.0545
Alcohol drinkers (%) **	3651	2198 (60.2%)	−0.2118
Diabetes (%) **	5120	286 (5.6%)	−0.0659
History of pancreatic disease (%) **	4766	62 (1.3%)	−0.0327
WBC (×10^3^/μL)	5102	4.8 (4.1–5.8)	−0.0468
RBC (×10^4^/μL)	5101	458.0 (427.0–489.0)	0.0784
Hb (g/mL)	5101	14.1 (13.1–15.1)	0.1273
Ht (%)	5101	42.3 (39.7–45.1)	0.1008
PLT (/μL)	5064	23.2 (20.1–27.0)	0.1263
TP (g/dL)	4716	7.20 (6.95–7.40)	0.0641
ALB (g/dL)	4240	4.4 (4.2–4.6)	0.1378
AST (U/L)	5099	21.0 (18.0–25.0)	0.1344
ALT (U/L)	5100	19.0 (14.0–26.0)	0.0964
ALP (U/L)	4670	185.0 (153.0–225.0)	−0.0014
γ-GTP (U/L)	5043	25.0 (17.0–41.0)	0.1890
T.BIL (mg/dL)	4457	0.8 (0.7–1.1)	0.0345
UA (mg/dL)	5092	5.3 (4.3–6.3)	0.0957
BUN (mg/dL)	4639	13.0 (11.0–15.0)	−0.0672
Cre (mg/dL)	5093	0.74 (0.63–0.86)	−0.0017
Na (mmol/L)	4128	140.0 (139.0–142.0)	−0.0166
K (mmol/L)	4128	4.1 (3.9–4.3)	0.0330
CL (mmol/L)	4128	104.0 (102.0–105.0)	−0.0893
Glucose (mg/dL)	5099	96.0 (90.0–103.0)	0.0540
HbA1c (%)	4568	5.5 (5.3–5.7)	−0.0380
TC (mg/dL)	5021	206.0 (185.0–229.0)	0.2498
LDL (mg/dL)	5100	123.0 (104.0–143.0)	0.1234
HDL (mg/dL)	5100	66.0 (55.0–79.0)	0.2428
TG (mg/dL)	5100	85.0 (60.0–123.0)	0.0919
Amy (U/L)	4903	72.0 (58.0–89.0)	−0.0376
Pancreatic lesions detected by ultrasonography (%) **	4675	151 (3.2%)	−0.0601

Data are shown as median and interquartile range. * Data are shown as median and range values. ** Data are shown as absolute values and percentages. Spearman’s correlation coefficients were calculated for the relationship between each background characteristic and the plasma apoA2-ATQ/AT level.

**Table 2:** Comparison of background characteristics between apoA2-ATQ/AT-positive and -negative groups in the screening study.

Covariates	Negative Group (apoA2-ATQ/AT: >35 μg/mL) (*n* = 5036)			Positive Group (apoA2-ATQ/AT: ≤35 μg/mL) (*n* = 84)			Univariate Analysis (*n* = 5120)				Multivariate Analysis (*n* = 3540)			
	*n*	Values		*n*	Values		OR	95% CI		*P*	OR	95% CI		*P*
Age (years) *	5024	52.0	(22–89]	84	61.0	(30–88)	1.76	1.46	2.11	<0.001	1.37	1.01	1.85	0.041
Males (%) **	5024	2607	(51.9)	84	55	(65.5)	0.57	0.36	0.89	0.015				
Height (cm)	4419	164.9	(158.3–171.5)	67	166.8	(158.6–171.3)	1.01	0.98	1.04	0.515				
Weight (kg)	4427	61.9	(53.2–70.8)	68	66.4	(54.3–75.7)	1.02	1.00	1.04	0.017				
BMI (kg/m2)	4419	22.5	(20.5–24.8)	67	23.5	(21.1–26.4)	1.09	1.02	1.16	0.010				
Smoking (%) **	3979	1526	(38.4)	42	22	(52.4)	1.77	0.96	3.25	0.067				
Alcohol consumption (%) **	3610			41										
Daily		831	(23.0)		4	(9.8)	0.46	0.11	1.84	0.271				
A few times a week		1347	(37.3)		16	(39.0)	1.13	0.38	3.40	0.830				
Once a month		1052	(29.1)		17	(41.5)	1.54	0.51	4.59	0.443				
Never		380	(10.5)		4	(9.8)	1.00	–	–	–				
Diabetes (%) **	5036	267	(5.3)	84	19	(22.6)	5.22	3.09	8.83	<0.001	2.00	0.74	5.44	0.174
History of pancreatic disease (%) **	4689	57	(1.2)	77	5	(6.5)	5.64	2.2	14.5	<0.001	2.91	0.73	11.57	0.130
WBC (×103/μL)	5018	4.8	(4.1–5.8)	84	5.8	(4.8–7.0)	1.38	1.25	1.52	<0.001	1.32	1.11	1.56	0.001
RBC (×104/μL)	5017	458.0	(427.0–489.0)	84	444.0	(409.5–489.0)	1.00	0.99	1.00	0.077				
Hb (g/mL)	5017	14.1	(13.1–15.1)	84	13.9	(12.5–14.9)	0.88	0.77	1.01	0.080				
Ht (%)	5017	42.3	(39.7–45.1)	84	41.5	(38.0–45.5)	0.95	0.9	1.00	0.047				
Plt (/μL)	4980	23.2	(20.1–27.0)	84	21.9	(18.6–25.9)	0.97	0.93	1.01	0.180				
TP (g/dL)	4633	7.2	(7.0–7.4)	83	7.1	(6.7–7.4)	0.80	0.5	1.29	0.358				
ALB (g/dL)	4170	4.4	(4.2–4.6)	70	4.3	(4.0–4.5)	0.70	0.42	1.16	0.165				
AST (U/L)	5015	21.0	(18.0–25.0)	84	21.0	(17.0–25.0)	1.00	0.99	1.02	0.758				
ALT (U/L)	5016	19.0	(14.0–26.0)	84	18.0	(14.0–27.0)	1.00	0.99	1.01	0.972				
ALP (U/L)	4588	185.0	(153.0–224.0)	82	198.0	(158.0–243.0)	1.00	1.00	1.01	0.029				
γ-GTP (U/L)	4960	25.0	(17.0–41.0)	83	22.0	(15.0–39.0)	1.00	1.00	1.01	0.253				
T.BIL (mg/dL)	4383	0.8	(0.7–1.1)	74	0.8	(0.6–1.0)	0.27	0.14	0.54	<0.001	0.44	0.18	1.07	0.071
UA (mg/dL)	5008	5.3	(4.3–6.3)	84	5.4	(4.3–6.2)	1.01	0.86	1.17	0.945				
BUN (mg/dL)	4557	13.0	(11.0–15.0)	82	15.0	(12.0–18.0)	1.09	1.05	1.14	<0.001	1.04	0.96	1.13	0.287
Cre (mg/dL)	5009	0.74	(0.63–0.86)	84	0.76	(0.66–0.87)	2.23	1.18	4.21	0.014				
Na (mmol/L)	4050	140.0	(139.0–142.0)	78	141.0	(140.0–142.0)	1.10	0.97	1.24	0.144				
K (mmol/L)	4050	4.1	(3.9–4.3)	78	4.1	(3.8–4.3)	0.86	0.42	1.76	0.675				
CL (mmol/L)	4050	104.0	(102.0–105.0)	78	104.0	(102.0–106.0)	0.96	0.87	1.07	0.468				
Glucose (mg/dL)	5015	96.0	(90.0–103.0)	84	100.0	(91.5–113.5)	1.02	1.01	1.03	<0.001	0.99	0.97	1.01	0.397
HbA1c (%)	4501	5.5	(5.3–5.7)	67	5.6	(5.3–6.4)	1.82	1.46	2.28	<0.001	1.12	0.65	1.94	0.684
TC (mg/dL)	4937	206.0	(185.0–229.0)	84	184.5	(160.5–211.0)	0.98	0.97	0.99	<0.001	0.94	0.92	0.97	<0.001
LDL (mg/dL)	5016	123.0	(104.0–144.0)	84	118.0	(90.5–133.0)	0.99	0.98	0.99	<0.001	1.05	1.01	1.08	0.006
HDL (mg/dL)	5016	66.0	(55.0–79.0)	84	54.0	(47.5–67.0)	0.96	0.94	0.97	<0.001	1.00	0.97	1.03	0.805
TG (mg/dL)	5016	85.0	(60.0–123.0)	84	82.0	(58.5–111.5)	1.00	0.99	1.00	0.300				
Amy (U/L)	4822	72.0	(58.0–89.0)	81	67.0	(52.0–87.0)	1.00	0.99	1.01	0.544				
Pancreatic lesions detected by Ultrasonography (%) **	4600	141	(3.1)	75	10	(13.3)	4.87	2.45	9.67	<0.001	3.04	1.01	9.14	0.047

In univariate logistic regression analyses, patient characteristics were compared using Fisher’s exact test for categorical variables and Welch’s *t* test or the Wilcoxon rank-sum test for continuous variables. Factors that exhibited significance in univariate analyses were included in the multivariate logistic regression analysis. Statistical significance was defined as a value of *p* < 0.05. Data are shown as median and interquartile range. * Data are shown as median and range values, and odds ratios (ORs) per 10 years are estimated. ** Data are shown as absolute values and percentages. OR, odds ratio; 95% CI, 95% confidence interval; *p*, *p*-value.

**Table 3:** Background characteristics of subjects in the cross-sectional study.

Characteristics	Number
All	105
Age (years) *	69.0 (21–86)
Male (%) **	73 (69.5)
Diabetes (%) **	10 (9.5)
PC	41
Resectable	17
Stage IA	2
Stage IB	0
Stage IIA	11
Stage IIB	4
Unresectable	24
Stage III	11
Stage IV	13
High-risk diseases (HR)	37
PCL	24
IPMN	21
CP	13
Others	27
AIP	13
NET	6
SPN	1
Undefined pancreatic mass	1
Normal	6

PC was staged according to the UICC classification, 7th edition. * Data are shown as median and range values. ** Data are shown as absolute values and percentages. PC, pancreatic cancer; PCL, pancreatic cystic lesions; IPMN, intraductal papillary mucinous neoplasm; CP, chronic pancreatitis; AIP, autoimmune pancreatitis; NET, pancreatic neuroendocrine tumors; SPN, solid pseudo-papillary neoplasms.

**Table 4:** Sensitivity, specificity and AUC values obtained using various cut-off values.

Category	apoA2-ATQ/AT Cut-Off Value (μg/mL)	Original Estimate						Bias-Adjusted Estimate *^,1^		
		Se (%)	(95% CI)	Sp (%)	(95% CI)	AUC	(95% CI)	Se (%)	Sp (%)	AUC
PC (*n* = 41)	≤35.0	51.2	(35.1–67.1)	98.8	(98.4–99.1)	0.903	(0.851–0.955)	51.3	98.8	0.889
	≤40.0	61.0	(44.5–75.8)	96.5	(95.9–97.0)			61.5	96.5	
	≤45.4	73.2	(57.1–85.8)	90.5	(89.6–91.4)			72.0	90.6	
Resectable PC (*n* = 17)	≤35.0	41.2	(18.4–67.1)	98.8	(98.4–99.1)	0.864	(0.765–0.963)	40.6	98.9	0.845
	≤40.0	47.1	(23.0–72.2)	96.5	(95.9–97.0)			47.5	96.6	
	≤45.4	64.7	(38.3–85.8)	90.5	(89.6–91.4)			61.0	90.6	
Unresectable PC (*n* = 24)	≤35.0	58.3	(36.6–77.9)	98.8	(98.4–99.1)	0.930	(0.877–0.983)	58.2	98.8	0.892
	≤40.0	70.8	(48.9–87.4)	96.5	(95.9–97.0)			71.0	96.6	
	≤45.4	79.2	(57.8–92.9)	90.5	(89.6–91.4)			79.0	90.7	
PCL (*n* = 24)	≤35.0	20.8	(7.1–42.2)	98.8	(98.4–99.1)	0.782	(0.675–0.889)	19.7	98.8	0.767
	≤40.0	33.3	(15.6–55.3)	96.5	(95.9–97.0)			30.1	96.6	
	≤45.4	58.3	(36.6–77.9)	90.5	(89.6–91.4)			49.6	90.9	
CP (*n* = 13)	≤35.0	53.8	(25.1–80.8)	98.8	(98.4–99.1)	0.871	(0.722–1.000)	51.3	98.9	0.863
	≤40.0	69.2	(38.6–90.9)	96.5	(95.9–97.0)			65.7	96.7	
	≤45.4	76.9	(46.2–95.0)	90.5	(89.6–91.4)			73.5	90.7	

A combination of subjects from the experimental prospective screening study and the cross-sectional study was used for this analysis. PC, pancreatic cancer; PCL, pancreatic cystic lesions; CP, chronic pancreatitis; Se, sensitivity; Sp, specificity; AUC, area under the ROC curve. *^,1^ The 0.632+ bootstrap method was used.

## Supplementary Materials

The following are available online at https://www.mdpi.com/2072-6694/12/9/2625/s1, Figure S1: Distribution of plasma apoA2-ATQ/AT levels among all subjects in the screening study, Figure S2: Distribution of plasma apoA2-ATQ/AT levels in the cross-sectional study, Table S1: Comparison of background characteristics between subjects in the apoA2-ATQ/AT-positive group who did and did not undergo imaging examinations, Table S2: Final diagnoses obtained using each imaging modality in the screening study, Table S3: Positive predictive value of the plasma apoA2-ATQ/AT level for detecting pancreatic cancer or high-risk lesions in patients with or without abnormal pancreatic ultrasonographic findings, Table S4: Sensitivity values obtained using various cut-off values in the cross-sectional study.
