# Supplementary material for: Prospective Study Using Plasma Apolipoprotein A2-Isoforms to Screen for High-Risk Status of Pancreatic Cancer
Source: Cancers (Basel). 2020 Sep 14;12(9):2625. doi: 10.3390/cancers12092625 (PMC7564617; doi:10.3390/cancers12092625)
Supplement: Supplementary file 1 [file cancers-12-02625-s001.pdf]

Supplemental table 1. Comparison of background characteristics between subjects in the apoA2-ATQ/AT-positive group who did and did not undergo imaging examinations

Characteristics	No imaging examination performed (*n* = 30)			Imaging examination performed (*n* = 54)			P-value
	n	Values		n	Values		
Age (years)*	30	58.0	(30–88)	54	63.5	(32–88)	0.119
Males (%)**	30	21	(70)	54	34	(63)	0.634
Height (cm)	26	167.7	(158.0–171.3)	41	166.2	(159.2–171.3)	0.857
Weight (kg)	26	63.8	(52.3–76.7)	42	67.6	(55.2–74.0)	0.696
BMI (kg/m^2)	26	22.4	(20.2–26.9)	41	24.5	(22.0–26.3)	0.385
Smokers (%)**	17	9	(52.9)	25	13	(52)	1
Alcohol consumption (%)	17			24			0.529
daily		1	(5.9)		3	(12.5)	
a few times a week		7	(41.2)		9	(37.5)	
once a month		6	(35.3)		11	(45.8)	
never		3	(17.6)		1	(4.2)	
Diabetes (%)**	30	8	(26.7)	54	11	(20.4)	0.589
History of pancreatic disease (%)**	29	0	(0)	48	5	(10.4)	0.15
WBC (×10/μL)	30	6.3	(5.0–6.8)	54	5.4	(4.6–7.3)	0.092
RBC (×10/μL)	30	456.5	(407.0–496.0)	54	439.0	(410.0–484.0)	0.393
Hb (g/mL)	30	14.3	(12.7–15.1)	54	13.5	(12.0–15.0)	0.126
Ht (%)	30	43.0	(38.5–45.8)	54	41.1	(37.3–44.3)	0.206
Plt (/μL)	30	22.6	(18.7–27.0)	54	21.7	(18.1–25.8)	0.421
TP (g/dL)	30	7.1	(6.7–7.3)	53	7.1	(6.8–7.5)	0.497
ALB (g/dL)	22	4.3	(4.1–4.5)	48	4.3	(4.0–4.5)	0.922
AST (U/L)	30	19.0	(14.0–23.0)	54	22.0	(18.0–27.0)	0.007
ALT (U/L)	30	17.0	(13.0–23.0)	54	19.0	(14.0–33.0)	0.308
ALP (U/L)	29	200.0	(164.0–235.0)	53	198.0	(155.0–253.0)	0.965
γ-GTP (U/L)	29	23.0	(17.0–36.0)	54	21.0	(15.0–40.0)	0.947
T.BIL (mg/dL)	28	0.8	(0.6–1.1)	46	0.8	(0.6–1.0)	0.258
UA (mg/dL)	30	5.5	(4.6–6.3)	54	5.3	(4.3–6.2)	0.818
BUN (mg/dL)	30	14.5	(11.0–18.0)	52	15.0	(12.0–18.0)	0.889
Cre (mg/dL)	30	0.79	(0.68–0.87)	54	0.75	(0.65–0.85)	0.761
Na (mmol/L)	29	140.0	(140.0–141.0)	49	141.0	(140.0–142.0)	0.314
K (mmol/L)	29	4.2	(3.8–4.4)	49	4.0	(3.9–4.3)	0.452
Cl (mmol/L)	29	104.0	(102.0–106.0)	49	103.0	(102.0–105.0)	0.536
Glucose (mg/dL)	30	102.5	(97.0–114.0)	54	97.0	(86.0–109.0)	0.079
HbA1c (%)	28	5.5	(5.3–6.4)	39	5.6	(5.4–6.4)	0.426
TC (mg/dL)	30	182.0	(168.0–214.0)	54	187.5	(160.0–211.0)	0.907
LDL (mg/dL)	30	121.0	(90.0–141.0)	54	115.0	(91.0–133.0)	0.383
HDL (mg/dL)	30	52.0	(45.0–66.0)	54	55.5	(49.0–67.0)	0.191
TG (mg/dL)	30	92.0	(66.0–121.0)	54	80.5	(55.0–105.0)	0.193
Amy (U/L)	29	69.0	(52.0–90.0)	52	65.5	(53.0–86.0)	0.906
Pancreatic lesions detected by ultrasonography (%)**	28	1	(3.6)	47	9	(19.1)	0.08

In univariate logistic analyses, patient characteristics were compared using Fisher’s

exact test for categorical variables, and Welch’s t test or the Wilcoxon rank-sum test for continuous variables. Values of $p<0.05$ were considered statistically significant.

Data are shown as median and interquartile range values. *Data are shown as median and range values. **Data are shown as absolute values and percentages. P, probability

Supplemental table 2. Final diagnoses obtained using each imaging modality in the screening study

	Total	CECT	MRCP	EUS
Number	54	39	5	10
Pancreatic cancer	1	1	0	0
Pancreatic cystic lesions	14	8	3	3
Intraductal papillary mucinous neoplasms	9	6	3	0
Undefined pancreatic cysts	5	2	0	3
Chronic pancreatitis	3	1	0	2
Pancreatic neuroendocrine tumors	1	0	0	1
Autoimmune pancreatitis	1	1	0	0
History of pancreatoduodenectomy	1	1	0	0
Agenesis of body and tail of the pancreas	1	1	0	0
Undefined pancreatic masses	1	1	0	0
Focal fat invasion in the pancreas	1	0	1	0
Suspected early chronic pancreatitis	2	0	0	2
Normal pancreatic findings	28	25	1	2

CECT, contrast-enhanced computed tomography; MRCP, magnetic resonance cholangiopancreatography; EUS, endoscopic ultrasonography; IPMN, intraductal papillary mucinous neoplasms

Supplemental table 3. Positive predictive value of the plasma apoA2-ATQ/AT level for detecting pancreatic cancer or high-risk lesions in patients with or without abnormal pancreatic ultrasonographic findings

	All subjects with US findings	Abnormal pancreatic US findings	Normal pancreatic US findings
Number	4675	151	4524
apoA2-ATQ/AT-positive	75	10	65
Underwent imaging	47	9	38
PC	1	1	0
PCL (IPMN)	10 (6)	4 (4)	6 (2)
CP	2	0	2
PPV for PC, PCL, or CP	27.7% (13/47)	55.5% (5/9)	21.1% (8/38)

PC, pancreatic cancer; PCL, pancreatic cystic lesions; CP, chronic pancreatitis; PPV, positive predictive value; US, ultrasonography

Supplemental table 4. Sensitivity values obtained using various cut-off values in the cross-sectional study

Disease (number)	Sensitivity (apoA2-ATQ/AT level: ≤35.0 μg/mL)<br>Sensitivity (apoA2-ATQ/AT level: ≤40.0 μg/mL)<br>Sensitivity (apoA2-ATQ/AT level: ≤45.4 μg/mL)				
PC (n=41)	51.2%<br>61.0%<br>73.2%	R (n=17)	41.2%<br>47.1%<br>64.7%	IA (n=2)	0%<br>0%<br>50.0%
				IIA (n=11)	45.5%<br>54.5%<br>72.7%
				IIB (n=4)	50.0%<br>50.0%<br>50.0%
		UR (n=24)	58.3%<br>70.8%<br>79.2%	III (n=11)	54.5%<br>72.7%<br>81.8%
				IV (n=13)	61.5%<br>69.2%<br>76.9%

PC was staged according to the UICC classification, 7th edition.

PC, pancreatic cancer; R, resectable stages; UR, unresectable stages; PCL, pancreatic cystic lesions; CP, chronic pancreatitis

Supplemental figure 1. Distribution of plasma apoA2-ATQ/AT levels among all subjects in the screening study

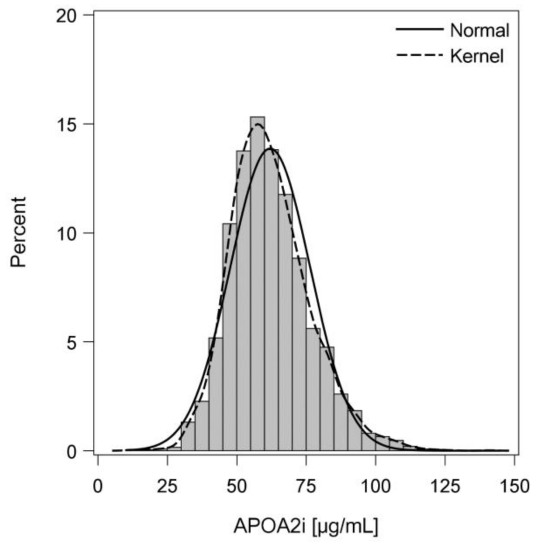

A Gray frequency distribution of the subjects' apoA2-ATQ/AT (µg/mL) levels is shown. The broken line indicates the Kernel density estimate, and the solid line shows the normal distribution. The mean (±SD) plasma apoA2-ATQ/AT level was 62.1 (±14.4) µg/mL.

apoA2-ATQ/AT, apolipoprotein A2-ATQ/AT

Supplemental figure 2. Distribution of plasma apoA2-ATQ/AT levels in the cross-sectional study

		Pancreatic cancer		High-risk disease		Normal-risk disease		Screening study
	All	Resectable	Unresectable	PCL	CP	AIP	NET	All
Number	105	17	24	24	13	13	6	5120
Mean apoA2-ATQ/AT (μg/mL)	37.6	37.5	34.8	45.9	35.1	24.1	46.5	62.1
Sensitivity (apoA2-ATQ/AT ≤35 μg/mL)		41.2%	58.3%	20.8%	53.8%	69.2%	0.0%	

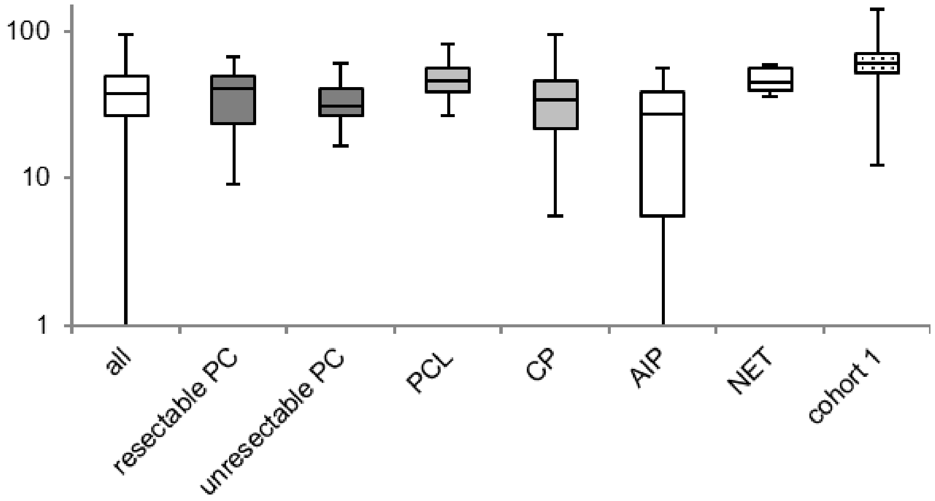

The numbers of subjects, mean apoA2-ATQ/AT levels, and sensitivity values obtained using a plasma apoA2-ATQ/AT level cut-off value of ≤35 μg/mL for each disease are shown in the upper table. The results of the screening study are also shown on the right side of the table for comparison. The distribution of plasma apoA2-ATQ/AT levels is shown in the lower panel. No data for solid pseudo-papillary neoplasms or undefined masses are shown. Cohort 1 included all of the samples in the screening study.

center line, median; box limits, upper and lower quartiles; whiskers, 1.5x interquartile

range; points, outliers

apoA2, apolipoprotein A2; PCL, pancreatic cystic lesions; CP, chronic pancreatitis; AIP, autoimmune pancreatitis; NET, pancreatic neuroendocrine tumors
